# Behavioural thermoregulation in cold‐water freshwater fish: Innate resilience to climate warming?

**DOI:** 10.1111/faf.12720

**Published:** 2022-11-29

**Authors:** Fatima Amat‐Trigo, Demetra Andreou, Phillipa K. Gillingham, J. Robert Britton

**Affiliations:** ^1^ Department of Life and Environmental Sciences, Faculty of Science and Technology Bournemouth University Poole UK

**Keywords:** climate change, latitude, microclimate use, salmonids, temperature difference, thermal refugia

## Abstract

Behavioural thermoregulation enables ectotherms to access habitats providing conditions within their temperature optima, especially in periods of extreme thermal conditions, through adjustments to their behaviours that provide a “whole‐body” response to temperature changes. Although freshwater fish have been detected as moving in response to temperature changes to access habitats that provide their thermal optima, there is a lack of integrative studies synthesising the extent to which this is driven by behaviour across different species and spatial scales. A quantitative global synthesis of behavioural thermoregulation in freshwater fish revealed that across 77 studies, behavioural thermoregulatory movements by fish were detected both vertically and horizontally, and from warm to cool waters and, occasionally, the converse. When fish moved from warm to cooler habitats, the extent of the temperature difference between these habitats decreased with increasing latitude, with juvenile and non‐migratory fishes tolerating greater temperature differences than adult and anadromous individuals. With most studies focused on assessing movements of cold‐water salmonids during summer periods, there remains an outstanding need for work on climatically vulnerable, non‐salmonid fishes to understand how these innate thermoregulatory behaviours could facilitate population persistence in warming conditions.

**Table:** 

1 INTRODUCTION	187
2 METHODS	188
2.1 Data collection	188
2.2 Data analysis	189
3 RESULTS	189
3.1 Extent and measurement of behavioural thermoregulation in freshwater fish	189
3.2 Temperature differences driven by behavioural thermoregulation	189
3.3 Cool‐water behavioural thermoregulation by salmonids	190
4 DISCUSSION	190
ACKNOWLEDGEMENTS	193
CONFLICT OF INTEREST	193
DATA AVAILABILITY STATEMENT	193
REFERENCES	193

## 1 INTRODUCTION

Temperature is a principal abiotic factor influencing the physiology and ecology of ectothermic organisms (Cossins & Bowler, 1987; Huey & Kingsolver, 1993). During periods of temperature change, ectotherms respond physiologically, including metabolic responses that maintain the functioning of key biological processes (Willmer et al., 2009). Many ectotherms also adapt their behaviour to better tolerate their thermal environment, where their ambient temperature determines whether a particular behaviour is expressed or which of a possible suite of behaviours is performed as the temperature changes (Abram et al., 2017). These behavioural changes represent an integrated, whole‐body response to temperature change requiring the coordination of multiple physiological responses (e.g. metabolic rate, digestive processes and immunity) (Dell et al., 2011).

The whole‐body responses of ectotherms to temperature changes include behavioural thermoregulation, where the sub‐optimal temperature experienced by the individual results in a behavioural response to move to a different, more optimal temperature, so altering their physiological functioning (Abram et al., 2017; Goniea et al., 2006; Ritter et al., 2020). Depending on the direction and extent of the ambient temperature shift, and the traits of the species, these behavioural responses can include shifts in habitat selection (Abram et al., 2017; Haesemeyer, 2020). Changes in habitat selection in freshwater fish driven by behavioural thermoregulation are reliant on the presence of a range of thermally heterogeneous micro‐ or meso‐habitats within a waterbody (Lennox et al., 2019). In periods of elevated temperatures in rivers, small areas of cooler water are often encountered around groundwater upwelling, providing thermal refugia where temperatures are reduced from ambient by up to 10°C (Hare et al., 2021; Kanno et al., 2014; Power et al., 1999). In ponds and lakes, thermoclines can result in the surface waters being relatively warm versus deeper areas, with bolder individuals of warm‐water species then accessing these warmer surface waters to bask (Nordahl et al., 2020). These aquatic thermal habitats are analogous to microclimates in terrestrial ecosystems (Gillingham et al., 2012), where at the landscape scale, terrestrial microclimates can provide ectotherms with access to a range of habitats with temperature differences of over 5°C at any given time, a difference similar to the extent of warming expected in the more extreme climate change projections (Suggitt et al., 2011).

Climate change is already driving extremely low flow and high‐temperature events in rivers around the world (Gudmundsson et al., 2021), so knowledge of behavioural thermoregulation in freshwater species is increasingly important. Fish populations are already responding to warming in open waters by shifting to new areas that now provide their thermal optima (Pörtner & Knust, 2007), such as Atlantic cod (*Gadus morhua*, Gadidae) shifting to more northerly and deeper waters in the North Sea (Engelhard et al., 2014). However, not all fish species and populations can respond in this manner, such as those with ranges restricted to river basins, whose populations are unable to move fast enough to track their climate niche (Comte & Grenouillet, 2013). Populations of some cold‐water riverine fishes could therefore be particularly vulnerable to climate‐change‐driven extirpations (Pörtner & Knust, 2007; Ruiz‐Navarro et al., 2016), although there is high uncertainty as to whether this extirpation risk is reduced by the interaction of their innate behaviours and the availability of thermal refugia.

The role of behavioural thermoregulation in facilitating freshwater fish to regulate their body temperatures has been studied in lakes (e.g. Armstrong et al., 2016; Biro, 1998), reservoirs (e.g. Encina et al., 2008; Hitt et al., 2017) and rivers (e.g. Chiaramonte et al., 2016; Ritter et al., 2020). Thermoregulatory movements have been detected both vertically (e.g. Nordahl et al., 2020; Tanaka et al., 2000) and longitudinally (e.g. Brewitt et al., 2017; White et al., 2019). These movements have been recorded in fish moving from cool to warm waters (Encina et al., 2008; Frechette et al., 2018) and the converse (Dobos et al., 2016; Kaya et al., 1977; Wilbur et al., 2020). There remains, however, a lack of integrative studies synthesising information on behavioural thermoregulation across different fish species and habitat types, and the extent to which these innate behaviours could reduce the vulnerability of populations to the adverse effects of climate warming. Here, we present a quantitative global synthesis of the thermoregulatory behaviour of freshwater fish across different habitats, including analyses of the direction and extent of the thermoregulation, and how this relates to a range of biotic and abiotic factors. We posit that (i) the direction and extent of behavioural thermoregulation in fish will be species, life‐stage, habitat and location specific and (ii) where cold‐water species express behavioural thermoregulation in warmer periods to access cooler waters, the extent of the difference between the ambient and the thermal refuge temperature will be comparable to the temperature differences predicted between current and future conditions in climate change projections.

## 2 METHODS

### 2.1 Data collection

Information on behavioural thermoregulation in fish was extracted from peer‐reviewed literature in the databases Web of Science and Scopus in January 2021. The following keywords and their synonyms were used in searches: thermal microhabitats, behavioural thermoregulation, warming water, climate change and fish (see Table S1 for search parameter details). Several search lists with different term combinations generated an initial list of 1501 studies. To avoid including studies of limited relevance, the search results were filtered in three sequential stages, removing irrelevant studies based on (i) title (792 studies retained), (ii) abstract (269 studies retained) and (iii) full text (111 studies retained). The criteria used in the filtering were to include only studies: (i) performed on freshwater fish species and (ii) where behavioural thermoregulation was the focus of the study (e.g. avoiding studies where chemicals affected fish thermoregulation or where thermoregulation was studied together with other physiological features).

The final list of 111 studies was then extensively reviewed based on their full text, removing those studies that were mainly on open marine habitats. The final list of studies used for subsequent analyses provided 77 studies (Table S2) completed between 1977 and 2020. These studies enabled information on 42 variables to be extracted, including 19 descriptive variables for characterizing the context of the studies, eight variables for testing the hypotheses and 15 variables that described the temperature and fish life‐stage data (Table S3). The eight variables used in the hypothesis testing were: latitude, altitude, family, species, life stage (juvenile/adult), migratory status (resident/anadromous), the direction of thermoregulation (cool‐warm/warm‐cool) and its extent (temperature difference between main habitat and thermal refuge) (Table S3). Not all of the 77 retained studies provided data for all of the variables. Correspondingly, where latitude or altitude data were missing, the GPS position coordinate data of the study area were extracted from Google Earth (https://earth.google.com), with elevation data extracted from a topographic map website (https://en‐gb.topographic‐map.com). If the study was completed at the catchment level, an intermediate localisation point was selected for the latitudinal and altitudinal data. Extracting the relevant temperature data was more challenging given the inherently high variability in how the temperature had been recorded between the retained studies, where the extracted data comprised of at least one of the following variables: mean habitat and thermal refuge temperature data, minimum and maximum temperature data (but not mean values), fish body temperature and the temperature difference between main habitat and thermal refuge. The lack of homogeneity in temperature records resulted in some gaps in these data and so not all of the extracted data could be used in each suite of analyses.

### 2.2 Data analysis

The initial analysis used a sign test to determine the differences in the number of studies reporting the direction of behavioural thermoregulation, where studies showing no behavioural thermoregulation were considered as the median (*m* = 0), cool‐warm movements were positive (p+ > *m*) and warm‐cool movements were negative (p− < *m*). Linear mixed‐effects models (LMMs) were then used to test the environmental effects on the temperature differences between the main habitat and the thermal refuge. First, the LMMs included the temperature difference as the response variable, latitude and altitude and their interaction as fixed effects and species as a random effect. To test whether the amplitude of the temperature difference between the main habitat and the thermal refuge was influenced by the maximum temperature of the main habitat, LMMs were also used, where the temperature difference was the response variable, maximum habitat temperature and life stages of species (juvenile‐adult) and their interaction were included as fixed effects, and species as a random effect. In these analyses, the species *Oncorhynchus mykiss* (Salmonidae) was divided into rainbow trout (resident) and steelhead (anadromous), depending on which form had been used in the study. When random effect variance was close to zero, linear regressions (sum of squares Type I) were built with the model. In all cases, the initial model was the full model, with model fitting processes then used to select the final model according to Akaike's information criterion (AIC), using the *step* (model, direction = “both”) function in R and selecting the most parsimonious model. Comparing temperature differences between the different life stages of species (juvenile‐adult) and their migration status (anadromous‐resident) used nonparametric Mann–Whitney *U*‐tests, as the assumptions of normality in temperature data were not met (Shapiro–Wilk test *p* < .05). All analyses were performed using R (v4.0.3 for Windows, www.r‐project.org), with the function “lmer” of “lme4” R package (Bates et al., 2015) and the function “rand” of “lmerTest” package (Kuznetsova et al., 2017).

## 3 RESULTS

### 3.1 Extent and measurement of behavioural thermoregulation in freshwater fish

The 77 retained studies provided 208 individual entries of evidence of behavioural thermoregulation in freshwater fish, with most completed in the northern hemisphere (97%; Table S2, Figure S1), especially in North America (Canada: 23%; USA: 62%). Although the studies covered 28 species across eight families, 84% were focused on species of the Salmonidae family, although other families included the Centrarchidae (7%) and Esocidae (2%) (Table S2). Most of the studies were completed in rivers (71%) (Tables S2 and S4).

The direction of behavioural thermoregulation was more commonly from warm‐cool rather than cool to warm (68% vs. 14%), with 18% of entries showing no evidence of behavioural shift to either warmer or cooler water (sign test, *p* < .001). Where behavioural thermoregulation was evident, 74% of the fish movements were horizontal and 24% were vertical. Most studies were completed in summer (85%) (fall 7%, spring 4% and winter 3%, laboratory studies not included). Where the data capture method had relied on tagging, radio data storage tags were used most frequently (36%), but with other methods (e.g. acoustic tags [12%], PIT tags [8%] and snorkel [24%]) also used frequently (Table S5). Water temperature data were mainly recorded using submersible temperature recorders (61%), thermometers and data probes (23%) (Table S6).

### 3.2 Temperature differences driven by behavioural thermoregulation

Across all studies (irrespective of the direction of temperature change), the differences in water temperatures between the main habitat(s) and new thermal habitat ranged between 0.4 and 8.0°C (median [25th–75th percentiles]: 3.8 [2.8–6.6°C]). For populations moving from cool to warm, the resulting temperature increase was between 0.6 and 7.0°C (1.5 [0.7–4.0°C]); for those moving from warm to cool, the temperature decrease was between −0.4 and −8.0°C (−4.5 [−3.0 to −6.6°C]).

There were 10 species that expressed behavioural thermoregulation in both directions (Figure S2); some of these differed by season (e.g. Smallmouth bass [*Micropterus dolomieu*, Centrarchidae], Largemouth bass [*Micropterus salmoides*, Centrarchidae], Brown trout [*Salmo trutta*, Salmonidae] or Brook trout [*Salvenius fontinalis*, Salmonidae]), but others showed movements in both directions in summer (e.g. Common carp [*Cyprinus carpio*, Cyprinidae], Northern pike [*Esox lucius*, Esocidae], Atlantic salmon [*Salmo salar*, Salmonidae], Coho salmon [*Oncorhynchus kisutch*, Salmonidae], Rainbow trout [*Oncorhynchus mykiss*, Salmonidae] or Chinook salmon [*Oncorhynchus tshawytscha*, Salmonidae]) (Figure S2). In general, accessing warmer habitats was achieved by sun basking in ponds and using small areas of warmer water in rivers, whereas the use of cooler water was achieved by fish accessing tributaries or tributary plumes, areas around springs, deep pools in rivers and areas of deeper water in lakes.

### 3.3 Cool‐water behavioural thermoregulation by salmonids

**FIGURE 1:**
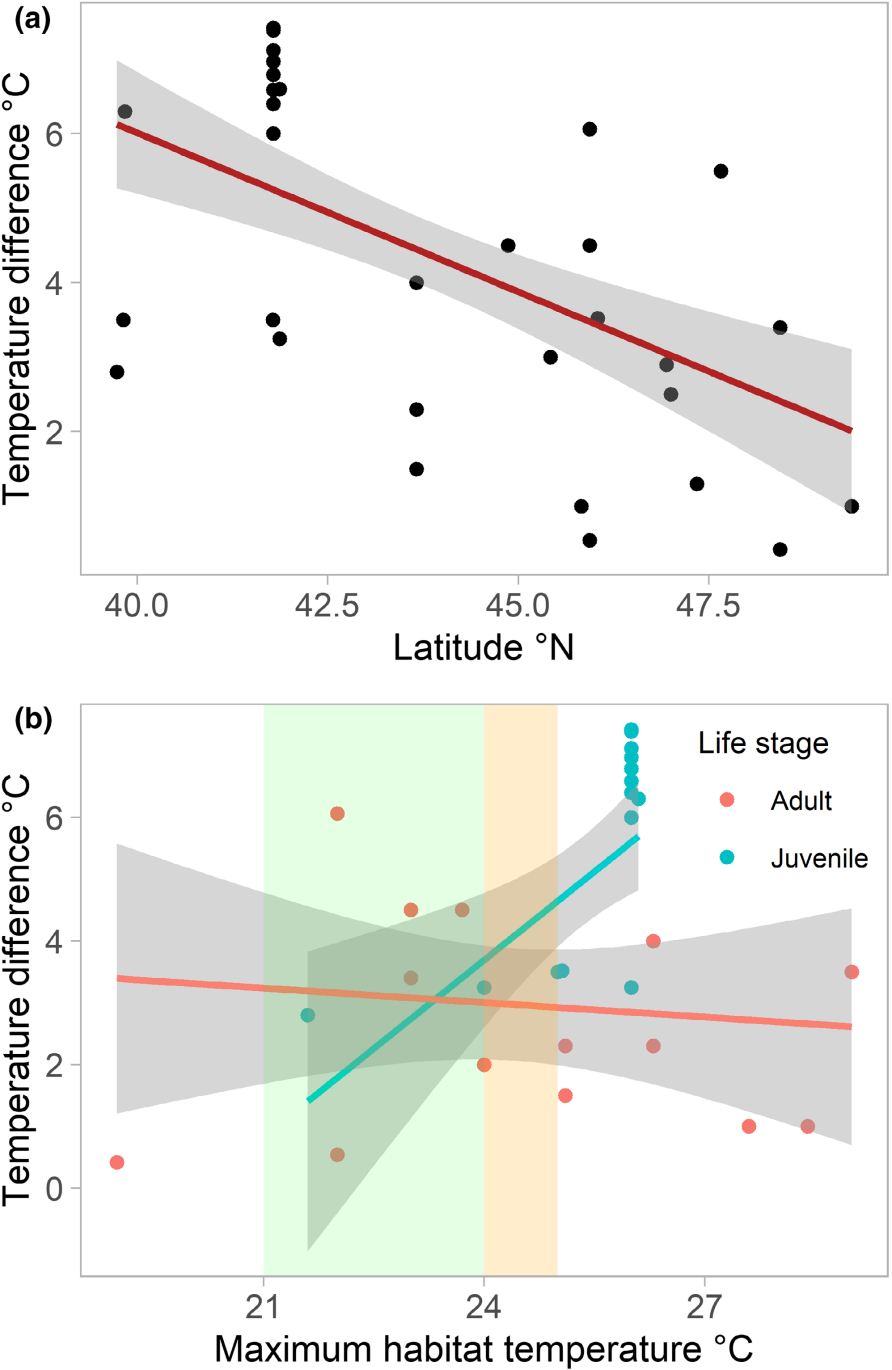
Relation between temperature differences between main habitat and refuge according to latitude (a) and maximum habitat temperature by life stage (b). In (b), blue and red lines represent data from juvenile and adult individuals respectively. The coloured rectangles represent the range of lethal temperature thresholds of steelhead (*Oncorhynchus mykiss*) (green) (21–24°C) and Chinook salmon (*Oncorhynchus tschawytscha*) (orange) (24–25°C) according to Carter (2008). Linear regression lines are shown with 95% confidence intervals indicated by shaded areas.

This dataset tested only observations of salmonid fishes shifting from warmer to cooler water in summer and was based on 63 observations from 22 studies, as other observations did not include the required information. Linear mixed models for temperature difference with latitude and altitude were found to be singular when species was used as a random effect (i.e. random effect variance was estimated near zero). During data exploration, two of the studies were found to be outliers and were removed from the successive linear regressions (Appendix S1), leaving 20 studies and 60 entries. The best‐fitting linear regression retained latitude as the only significant predictor of the temperature differences between the main and thermal refuge habitat, with the difference decreasing as latitude increased (*F*_(1,58)_ = 22.54, *p* < .001, *r* ^2^ = .28) (Table S7; Figure 1a). For the relationship between temperature differences and maximum temperatures in the main habitat by species (dataset including life stage information: 20 studies and 57 entries), the best‐fitting linear mixed‐effect model was significant only for juvenile fish, and the general pattern was that the warmer the original habitat, the greater the extent of temperature difference with the thermal refuge (Table 1, Table S8; Figure 1b).

**TABLE 1:** Results of linear mixed‐effects models relating variation in temperature difference to the maximum habitat temperature (Thabitatmax) and life stage (*p < .05)

Model	Fixed effects				Random effects (|Species) *SD*
	Coefficient	*SE*	df	*p*	
Intercept	−0.74	0.26	8.45	.02*	0.49
Thabitatmax	−0.13	0.39	29.67	.74	
Juvenile	−0.09	0.32	31.78	.78	
Thabitatmax: Juvenile	2.25	0.77	30.36	<.01*	

*Note*: The model with the lowest Akaike information criterion among all possible models is shown. Estimates of coefficients, standard error (*SE*) (fixed effects) and their standard deviation (*SD*) across species (random effects) are indicated.

**FIGURE 2:**
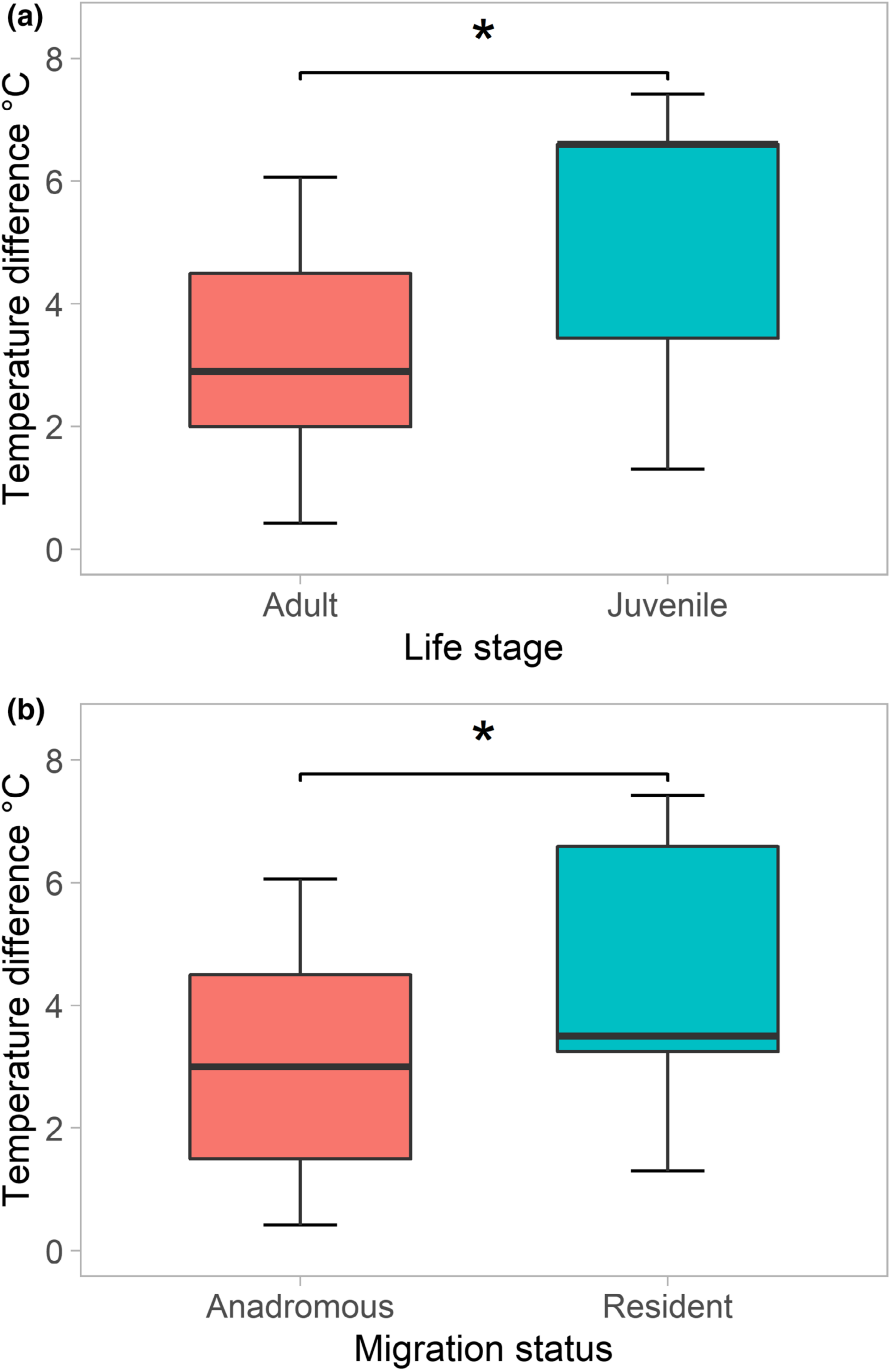
Box plots of temperature differences between the main habitat and refuge according to life stage (juvenile‐adult) of species (a) and anadromous versus resident fishes (b). The bottom and top of the boxes correspond to the first and third quartiles, bands represent the median and whiskers and circles represent the outlying values. “*” Indicates significant differences between groups according to Mann–Whitney *U*‐test (*p* < .05).

Comparisons of temperature differences between life stage and migratory status were completed on datasets comprising 20 studies (57 entries) and 17 studies (40 entries), respectively. They revealed movements of juvenile fish resulted in greater temperature differences than in adults (mean ± *SD*: −5.4 ± −1.8 vs. −3.2 ± −1.7°C; Mann–Whitney *U*‐test, *W* = 139, *p* < .001; Figure 2a), with this also the case between resident and anadromous species (−3.5 ± −1.9 vs. −3.0 ± −1.6°C; Mann–Whitney *U*‐test, *W* = 111, *p* = .017; Figure 2b).

## 4 DISCUSSION

In synthesising the global information on the behavioural thermoregulation of freshwater fish species over the past 40 years, we revealed a clear bias across completed studies; these were in geographic location (to North America), species (to salmonids) and habitats (to rivers). While testing our first hypothesis revealed movements in both temperature directions, studies were dominated by cold‐water fishes moving from warm to cool waters in summer (e.g. Chiaramonte et al., 2016; Goniea et al., 2006). Compared with adult and anadromous fishes, movements by juvenile and non‐migratory fishes resulted in a greater temperature difference between their original and thermal refuge habitats, with these temperature differences also higher at lower latitudes.

Increasing water temperatures through climate change are increasingly threatening cold‐water species with narrow thermal preferences, such as salmonid fishes (Myers et al., 2017). Temperature increases are leading to shifts in population distributions to cooler areas and the decline of some populations at local scales (Almodóvar et al., 2012; Comte & Grenouillet, 2013; Ruiz‐Navarro et al., 2016). However, such declines may proceed more slowly than previously thought due to the thermal stability of mountain streams which could mean cool refugia and the persistence of species that are able to access them (Isaak et al., 2016). As cold habitats decrease in their spatial extent then cold‐water refugia provide the opportunity for many species to maintain sustainable populations, but individuals might need to compete strongly to access these areas (Broadmeadow et al., 2011). We found that when fish moved from warm to cool habitats in rivers, the mean difference in water temperature between these habitats was 4.7°C. Greater differences in temperature between the habitats were reported in rainbow trout and brown trout moving to a tributary river, with cooler temperatures by up to 10°C in the tributary (Kaya et al., 1977). In the Klamath river, USA, juvenile Chinook salmon and Coho salmon occupied areas in a tributary river that were up to 8°C cooler than the main river (Chiaramonte et al., 2016). Models predict that for every 2°C increase in air temperature, there will be an increase in stream temperature of approximately 1.5°C, with this warming already being recorded in North American rivers and expected to occur in Europe (Hardenbicker et al., 2017; Isaak et al., 2017; Null et al., 2013). Our synthesis indicated that the deliberate movements of fish to cooler areas within thermally heterogeneous rivers can substantially reduce the water temperatures they experience—certainly more than the projected water temperature increases this century—indicating it could be a key mechanism for facilitating climatically vulnerable fish populations to persist in warming rivers (Nadeau et al., 2022).

The extent of temperature differences between the main river habitat and the thermal refuges used by fish were inversely related to latitude. Air temperatures generally decrease with increasing latitude and, consequently, water temperatures in streams and rivers also decrease (Harvey et al., 2011). Thus, as latitude increases, fish tend to experience lower temperatures in main river channels, resulting in smaller temperature differences between those habitats and cool‐water refugia. Latitude also influences the thermal characteristics of fishes generally, such as their critical thermal maximum (CTMax) (the temperature at which fish are sufficiently thermally stressed to lose their body equilibrium), which decreases with increasing latitude (Cereja, 2020; Nati et al., 2021). Although CTMax of species is determined by genetic adaptation, phenotypic plasticity facilitates some variability in maximum CTMax thresholds. Thus, intra‐specific variability in CTMax is also apparent with, for example, juvenile cutthroat trout (*Oncorhynchus clarkia pleuriticus*, Salmonidae) having elevated CTMax following acclimation periods at higher temperatures (e.g. CTmax of 24.6°C following acclimation to 10 vs. 29.4°C at 20°C) (Underwood et al., 2012). Experiments on juvenile Coho salmon revealed that population‐specific differences in CTMax were also due to different acclimation regimes rather than genetic adaptation (Konecki et al., 1995). These results on thermal plasticity suggest there is some capacity for local populations to respond to warming (Eliason et al., 2011; Stitt et al., 2014). Moreover, high intraspecific genetic variation across local populations could provide a higher potential for thermal acclimation in fish, which has been demonstrated in rainbow trout (Adams et al., 2022). This ability of some individuals to potentially acclimate to higher water temperatures can thus act in concert with behavioural thermoregulation to facilitate all thermal phenotypes of climatically vulnerable salmonid populations to persist in warming rivers. Notwithstanding, in lake trout (*Salvelinus namaycush*, Salmonidae), temperature acclimation of adult fish resulted in only limited trans‐generational plasticity to higher temperatures, suggesting this plasticity has limited potential to buffer warming effects on future generations (Penney et al., 2021). These limitations in adapting to higher temperatures have also been reflected in tropical fish species, with a higher risk that species already living in environments close to their upper thermal tolerance limit will be unable to respond sufficiently to expected rates of warming (Morgan et al., 2020).

The use of thermal refuges in fish can vary by body size (Brewitt et al., 2017; White et al., 2019; Wilbur et al., 2020), with our syntheses revealing that compared to adults, juvenile fish moved in a wider and higher temperature range between their main habitats and thermal refugia. These differences may be through adult fish having lower thermal tolerances and thus their movements to thermal refugia are triggered at lower water temperatures than in juveniles (Breau et al., 2007, 2011). Also, elevated temperatures increase metabolic demands on individuals (Norin & Clark, 2016), with fish body sizes positively correlated with metabolic demand, resulting in higher physiological stress in adults than juveniles as temperatures increase, forcing movements at lower temperature thresholds (Woodward et al., 2010). An alternative hypothesis is that larger individuals have a greater probability of being socially dominant and so are more actively accessing and then defending suitable refugia (Biro, 1998; Ebersole et al., 2001). In doing so, they potentially displace smaller individuals to suboptimal thermal areas (Ebersole et al., 2001; Ritter et al., 2020). Nevertheless, refuge occupancy by individuals is associated with trade‐offs with, for example, their foraging, predator avoidance and energy conservation during elevated flows (McCullough et al., 2009; White et al., 2019).

In addition, factors such as habitat structure, prey availability and/or the presence of parasites also influence the selection of habitats that might vary in their temperatures (Bevelhimer, 1996; Horký et al., 2019). For example, juvenile Coho salmon forage on sockeye salmon (*Oncorhynchus nerka*, Salmonidae) eggs that are only available in cold water during the summer, and then move into warmer waters to increase their digestive capacity (Armstrong et al., 2013; Armstrong & Schindler, 2013). Brewitt et al. (2017) revealed that larger steelhead juveniles were holding stations along the edge of thermal refuges where the optimal balance was achieved between temperature regulation and prey availability. Our synthesis also revealed this pattern in temperature difference was apparent between anadromous and non‐migratory fishes, where the latter experienced a wider range of temperatures that were also higher. In addition to anadromous fishes generally being larger, they are likely to have a greater capacity for movement, enabling them to access areas of thermal refuges during their migrations, whereas non‐migrants might lack access to thermal refugia in their immediate areas, resulting in less opportunity to access cooler waters (Dobos et al., 2016; Keefer et al., 2018). Some species such as Chinook salmon even alter their migration routes and timings when water temperatures exceed 20°C, with the increased use of cooler tributaries in warmer years (Goniea et al., 2006).

The focus of most of our synthesised studies on salmonid fishes is likely through the interaction of their relatively low thermal optima and the predicted temperature increases through climate change (Almodóvar et al., 2012; Ruiz‐Navarro et al., 2016). Populations of salmonid fishes are also considered culturally important and tend to have both high economic and recreational values in the global north (Briton, 2014; Raby et al., 2012). For example, economic estimates of the benefit of recreational fishing range from $148,000 to $713,000 per year for California golden trout (*Oncorhynchus mykiss aguabonita*, Salmonidae) (Alkire, 2003). This bias towards a greater number of studies on riverine salmonids is the likely reason that a higher number of studies reported horizontal movements during the summer where the direction of behavioural thermoregulation was towards cold water. However, many non‐salmonid species are also potentially going to be imperilled by climate change effects that are acting to both elevate instream temperatures and reduce flow rates (Gudmundsson et al., 2021).

The use of our specific search terms to enable quantitative analyses meant that some studies that incorporate aspects of behavioural thermoregulation were not included, such as some studies on habitat selection and use. These “missed” studies were on species including Northern pike (Pierce et al., 2013; Říha et al., 2021) and some salmonid fishes (e.g. Gutowsky et al., 2017). Thus, future work can place more emphasis on concepts of behavioural thermoregulation. Our quantitative analysis demonstrated there is an urgent need to understand how behavioural thermoregulation will influence the population persistence of a wider range of species and across more of the world's regions. These studies will be critical from a management and policy perspective, as they will show whether there is widespread adoption of behavioural thermoregulation as a mechanism to buffer populations from the adverse effects of warming. If so, then these studies will indicate the need for rivers to be thermally heterogeneous and provide access to a wide range of thermal habitats for fish to adapt to climate change in situ. Moreover, it is already clear that ensuring connectivity between the different thermal environments of rivers is essential since access to warm areas is also an important process for the development and survival of species (Armstrong et al., 2021). However, maintaining longitudinal connectivity is contrary to many river management practices around the world, where river engineering schemes (e.g. for hydropower, flood defence and navigation) are acting in the opposite direction. For example, barriers that impede fish movements in European rivers are present, on average, every 1.4 km (Belletti et al., 2020), with 99% of rivers in Britain being impacted by artificial barriers (Jones et al., 2019). This connectivity loss, coupled with warmer temperatures within the created lentic areas that further increase river temperatures downstream (Krztoń et al., 2022), all act contrary to the need to minimise instream temperatures and enable free movements of fish. Consequently, enabling climatically vulnerable fish populations to persist in future will require substantial changes in the prevailing management regimes of many of the world's river systems.

## CONFLICT OF INTEREST

The authors declare no conflicts of interest.

## Supporting information


Appendix S1

## Data Availability

No empirical data were used for this research. The data that support the findings of this study are openly available in [BORDaR] at http://bordar.bournemouth.ac.uk, reference name [THERMOS_Literature_Review_V1].
