# Supplementary material for: Behavioural thermoregulation in cold‐water freshwater fish: Innate resilience to climate warming?
Source: Fish Fish (Oxf). 2022 Nov 29;24(1):187–95. doi: 10.1111/faf.12720 (PMC10100141; doi:10.1111/faf.12720)
Supplement: Supplementary file 1 — Appendix S1 [file FAF-24-187-s001.docx]

Supplementary Information for

**Behavioural thermoregulation in cold-water freshwater fish: innate resilience to climate warming?**

Fatima Amat-Trigo, Demetra Andreou, Phillipa K. Gillingham and J. Robert Britton


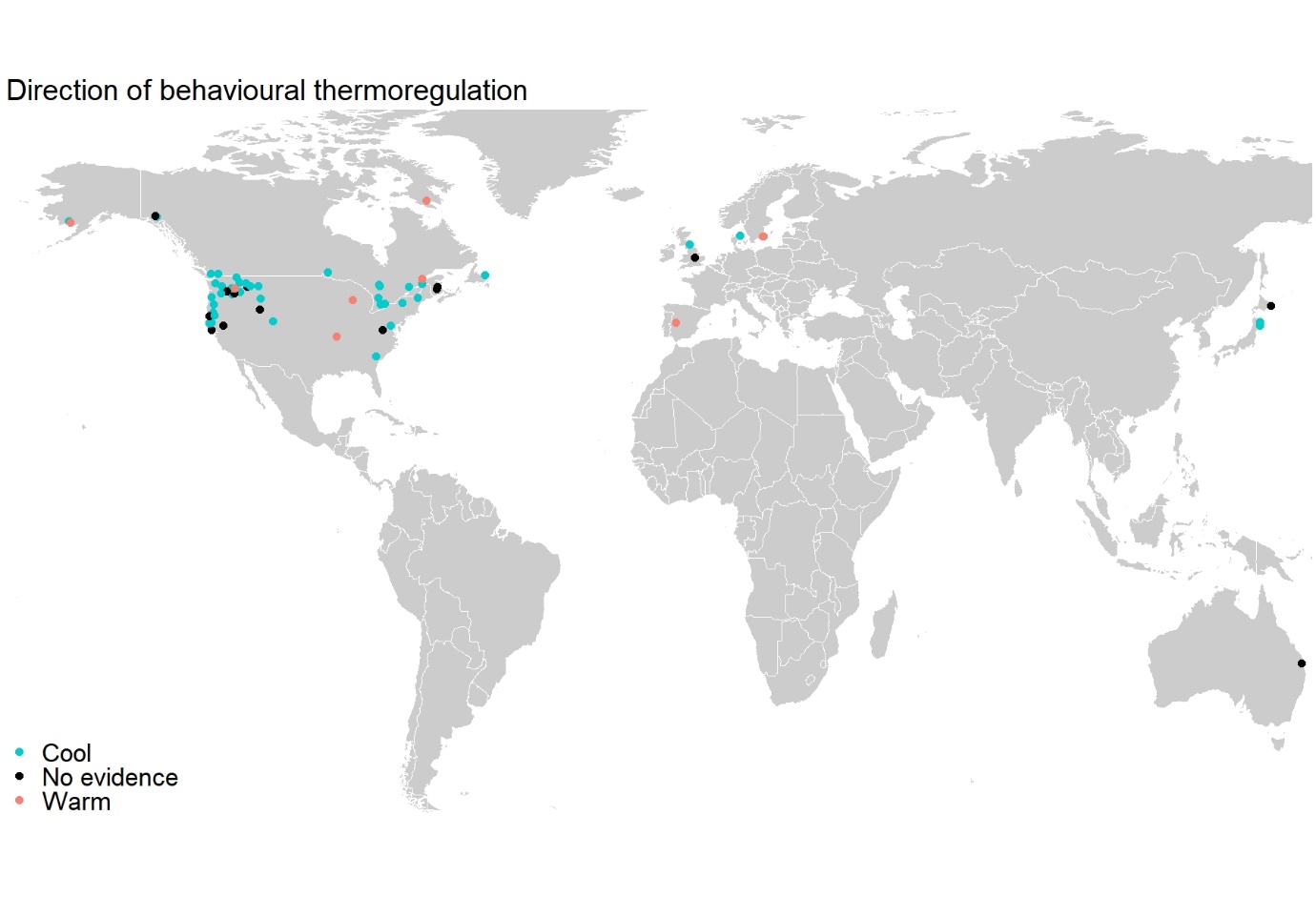


**Figure S1.** Geographical distribution of the database entries compiled by country. The color of the points shows the direction of behavioural thermoregulation from warmer to colder waters (blue), from colder to warmer waters (red) and no evidence of behavioural thermoregulation in the studies (black).


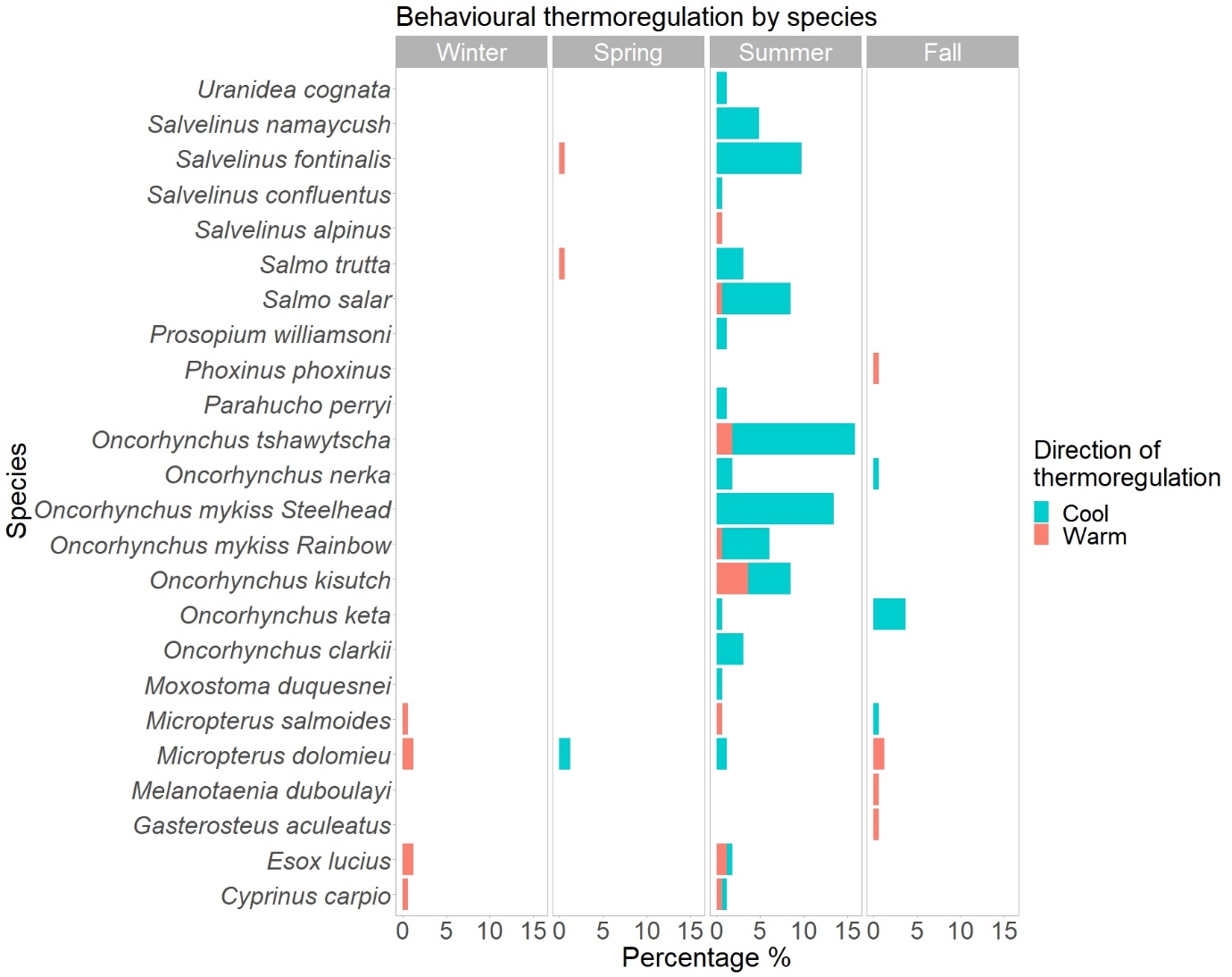


**Figure S2.** Percentage of studies in which species showed behavioural thermoregulation from warmer to colder waters (blue), from colder to warmer waters (red) or both by seasons.

**Table S1.** Parameters used for the literature search.

| **Parameters** | **Values** |
| --- | --- |
| Electronic datasets | Web of Science and SCOPUS |
| Publication time range | January 2000–December 2020 |
| Title | (fish) |
| Search parameters (1) topic (title OR keywords OR abstract) | (thermoregulation behavio*),  (thermoregulation (behavio* OR habit* OR conduct)),  (thermoregulation (behavio* OR demean*)),  (thermoregulation (behavio* OR demean* OR habit* OR conduct)) |
| Search parameters (2) topic (title OR keywords OR abstract) | (Climat* warm* OR Climat* change),  (global warm* OR global change),  (Climat* alteration),  (Climat* variation),  (Climat* NEAR/5 variation),  (warm* water),  (warm* (water OR river OR stream)),  (water (warm* OR heat* OR temperate)),  (Thermal (microhabitat OR niche OR range)),  (microhabitat (warm* OR hot)) |
|  |  |

**Table S2.** List of references used in the study. Information about latitude, altitude, country, family, species, season, habitat, direction of thermoregulation (Direction), maximum temperature of main habitat (Max. habitat temperature), temperature difference between main habitat and thermal refuge (Temp. difference), migratory status and life stage is given.

| **Reference** | **Latitude** | **Altitude** | **Country** | **Family** | **Species** | **Season** | **Habitat** | **Direction** | **Max. habitat temperature** | **Temp. difference** | **Migratory status** | **Life stage** |
| --- | --- | --- | --- | --- | --- | --- | --- | --- | --- | --- | --- | --- |
| Adams *et al.*, 2015 | 46.86176 | 945 | USA | Cottidae | *Uranidea cognata* | Summer | River | Cool | 18.4 |  | Resident |  |
|  | 46.86176 | 945 | USA | Cottidae | *Uranidea cognata* | Summer | River | Cool | 21.2 |  | Resident |  |
|  | 46.86176 | 945 | USA | Cottidae | *Uranidea bairdii* | Summer | River | No evidence | 18.4 |  | Resident |  |
|  | 46.86176 | 945 | USA | Cottidae | *Uranidea bairdii* | Summer | River | No evidence | 21.2 |  | Resident |  |
| Armstrong *et al.*, 2013 | 59.29559 | 7 | USA | Salmonidae | *Oncorhynchus kisutch* | Summer | River | Warm | 7.4 |  | Resident | Juvenile |
|  | 59.29559 | 7 | USA | Salmonidae | *Oncorhynchus kisutch* | Summer | River | Warm | 7.0 |  | Resident | Juvenile |
| Armstrong and Schindler, 2013 | 59.29559 | 7 | USA | Salmonidae | *Oncorhynchus kisutch* | Summer | River | Warm | 8.0 | 2.00 | Resident | Juvenile |
| Armstrong *et al.*, 2016 | 59.58144 | 18.29 | USA | Salmonidae | *Oncorhynchus nerka* | Summer | Lake | Cool | 19.9 |  | Anadromous | Adult |
| Baigun, 2003 | 43.39532 | 474.88 | USA | Salmonidae | *Oncorhynchus mykiss* (steelhead) | Summer | River | Cool | 22.0 |  | Anadromous | Adult |
| Baird and Krueger, 2003 | 43.66417 | 496.52 | USA | Salmonidae | *Salvelinus fontinalis* | Summer | River | Cool | 26.3 | 2.30 |  | Adult |
|  | 43.66417 | 496.52 | USA | Salmonidae | *Salvelinus fontinalis* | Summer | River | Cool | 26.3 | 4.00 |  | Adult |
|  | 43.66417 | 496.52 | USA | Salmonidae | *Oncorhynchus mykiss* (rainbow) | Summer | River | Cool | 25.1 | 1.50 |  | Adult |
|  | 43.66417 | 496.52 | USA | Salmonidae | *Oncorhynchus mykiss* (rainbow) | Summer | River | Cool | 25.1 | 2.30 |  | Adult |
| Baldock *et al.*, 2016 | 59.29559 | 7 | USA | Salmonidae | *Oncorhynchus kisutch* | Summer | River | Warm | 8.0 | 4.10 | Resident | Juvenile |
| Berman and Quinn, 1991 | 47.0026 | 478 | USA | Salmonidae | *Oncurhynchus tshawytscha* | Summer | River | Cool |  | 2.50 | Anadromous | Adult |
| Bertolo *et al.*, 2011 | 46.8026 | 389.84 | Canada | Salmonidae | *Salvelinus fontinalis* | Summer | Lake | Cool |  | 7.56 | Resident |  |
|  | 46.8026 | 389.84 | Canada | Salmonidae | *Salvelinus fontinalis* | Summer | Lake | Cool |  | 6.30 | Resident |  |
| Biro, 1998 | 44.75967 | 243 | Canada | Salmonidae | *Salvelinus fontinalis* | Spring | Lake | Warm | 18.5 |  | Resident | Juvenile |
|  | 44.75967 | 243 | Canada | Salmonidae | *Salvelinus fontinalis* | Summer | Lake | Cool |  |  | Resident | Juvenile |
| Birtwell *et al.*, 2003 | 49.34079 | 2 | Canada | Salmonidae | *Oncorhynchus keta* | Summer | Experiment | Cool | 24.1 |  | Resident | Juvenile |
| Block *et al.*, 1984 | 33.27676 | 63 | USA | Centrarchidae | *Micropterus salmoides* |  | Reservoir | Cool | 50 |  | Resident | Adult |
|  | 33.27676 | 63 | USA | Centrarchidae | *Lepomis macrochirus* |  | Reservoir | Cool | 50 |  | Resident | Adult |
| Breau *et al.*, 2007 | 46.87995 | 71.93 | Canada | Salmonidae | *Salmo salar* | Summer | River | No evidence |  |  | Resident | Juvenile |
|  | 46.87995 | 71.93 | Canada | Salmonidae | *Salmo salar* | Summer | River | Cool |  |  | Resident | Juvenile |
| Brewitt and Danner, 2014 | 41.78165 | 480.36 | USA | Salmonidae | *Oncorhynchus mykiss* (steelhead) | Summer | River | Cool | 26.0 | 6.97 | Resident | Juvenile |
|  | 41.78165 | 480.36 | USA | Salmonidae | *Oncorhynchus mykiss* (steelhead) | Summer | River | Cool | 26.0 | 7.42 | Resident | Juvenile |
|  | 41.78165 | 480.36 | USA | Salmonidae | *Oncorhynchus mykiss* (steelhead) | Summer | River | Cool | 26.0 | 6.00 | Resident | Juvenile |
|  | 41.78165 | 480.36 | USA | Salmonidae | *Oncorhynchus mykiss* (steelhead) | Summer | River | Cool | 26.0 | 7.39 | Resident | Juvenile |
|  | 41.78165 | 480.36 | USA | Salmonidae | *Oncorhynchus mykiss* (steelhead) | Summer | River | Cool | 26.0 | 7.12 | Resident | Juvenile |
|  | 41.78165 | 480.36 | USA | Salmonidae | *Oncorhynchus mykiss* (steelhead) | Summer | River | Cool | 26.0 | 6.59 | Resident | Juvenile |
|  | 41.78165 | 480.36 | USA | Salmonidae | *Oncorhynchus mykiss* (steelhead) | Summer | River | Cool | 26.0 | 6.40 | Resident | Juvenile |
|  | 41.78165 | 480.36 | USA | Salmonidae | *Oncorhynchus mykiss* (steelhead) | Summer | River | Cool | 26.0 | 6.79 | Resident | Juvenile |
| Brewitt *et al.*, 2017 | 41.77991 | 477.62 | USA | Salmonidae | *Oncorhynchus mykiss irideus* (steelhead) | Summer | River | Cool | 25.0 | 3.50 | Resident | Juvenile |
|  | 41.77991 | 477.62 | USA | Salmonidae | *Oncorhynchus tshawytscha* | Summer | River | Cool | 25.0 |  | Resident | Juvenile |
| Bunt *et al.*, 2013 | 43.40855 | 286.51 | Canada | Catostomidae | *Moxostoma duquesnei* | Summer | River | Cool |  |  | Resident | Juvenile |
| Caldwell *et al.*, 2020 | 41.2272 | 1657 | USA | Salmonidae | *Salvelinus fontinalis* |  | Lake | Cool |  |  | Resident | Adult |
| Chiaramonte and Ray, 2016 | 41.86937 | 543.76 | USA | Salmonidae | *Oncorhynchus tshawytscha* | Summer | River | Cool |  | 6.60 |  | Juvenile |
|  | 41.86937 | 543.76 | USA | Salmonidae | *Oncorhynchus kisutch* | Summer | River | Cool |  | 6.60 |  | Juvenile |
| Corey *et al.*, 2020 | 46.88215 | 64 | Canada | Salmonidae | *Salmo salar* | Summer | River | Cool |  |  | Resident | Juvenile |
|  | 46.88215 | 64 | Canada | Salmonidae | *Salmo salar* | Summer | River | No evidence |  |  | Resident | Juvenile |
|  | 46.88215 | 64 | Canada | Salmonidae | *Salmo salar* | Summer | River | Cool | 28.1 |  | Resident | Juvenile |
|  | 47.34755 | 137.16 | Canada | Salmonidae | *Salmo salar* | Summer | River | Cool | 30.3 |  | Resident | Juvenile |
|  | 47.34755 | 137.16 | Canada | Salmonidae | *Salmo salar* | Summer | River | No evidence |  |  | Resident | Juvenile |
|  | 47.34755 | 137.16 | Canada | Salmonidae | *Salmo salar* | Summer | River | Cool | 29.2 |  | Resident | Juvenile |
| Cote *et al.*, 2020 | 49.08698 | 42 | USA | Salmonidae | *Salvelinus fontinalis* | Summer | Lake | Cool |  |  | Resident | Adult |
| Dobos, 2016 | 45.82393 | 748.59 | USA | Salmonidae | *Oncorhynchus clarkii lewisi* | Summer | River | Cool | 28.4 | 1.00 | Anadromous | Adult |
|  | 45.82393 | 748.59 | USA | Salmonidae | *Oncorhynchus clarkii lewisi* | Summer | River | Cool | 27.6 | 1.00 | Anadromous | Adult |
| Donaldson *et al.*, 2009 | 49.3748 | 31.09 | Canada | Salmonidae | *Oncorhynchus nerka* | Summer | River | Cool |  | 1.00 | Anadromous | Adult |
| Dugdale *et al.*, 2016 | 47.34382 | 139.29 | Canada | Salmonidae | *Salmo salar* | Summer | River | Cool |  | 1.30 | Resident | Juvenile |
| Ebersole *et al.*, 2001 | 46.04088 | 373.99 | USA | Salmonidae | *Oncorhynchus mykiss* (rainbow) | Summer | River | Cool | 25.1 | 3.52 | Resident | Juvenile |
| Ebersole and Frissell, 2003 | 45.42322 | 817.17 | USA | Salmonidae | *Oncorhynchus mykiss* (rainbow) | Summer | River | Cool | 30.0 | 3.00 | Resident |  |
|  | 45.42322 | 817.17 | USA | Salmonidae | *Oncorhynchus tshawytscha* | Summer | River | Cool | 30.0 | 3.00 | Anadromous |  |
|  | 45.42322 | 817.17 | USA | Salmonidae | *Oncorhynchus mykiss* (rainbow) | Summer | River | Cool | 27.6 | 3.00 | Resident |  |
|  | 45.42322 | 817.17 | USA | Salmonidae | *Oncorhynchus tshawytscha* | Summer | River | Cool | 27.6 | 3.00 | Anadromous |  |
| Encina *et al.*, 2008 | 39.81667 | 253.29 | Spain | Cyprinidae | *Cyprinus carpio* | Summer | Reservoir | Cool |  | 7 | Resident |  |
|  | 39.81667 | 253.29 | Spain | Cyprinidae | *Cyprinus carpio* | Winter | Reservoir | Warm |  | 7 | Resident |  |
| Frechette *et al.*, 2018 | 48.43564 | 221.89 | Canada | Salmonidae | *Salmo salar* | Summer | River | Cool | 23.0 | 3.40 | Anadromous | Adult |
|  | 48.43564 | 221.89 | Canada | Salmonidae | *Salmo salar* | Summer | River | No evidence | 18.9 |  | Anadromous | Adult |
|  | 48.43564 | 221.89 | Canada | Salmonidae | *Salmo salar* | Summer | River | Warm | 16.9 | 2.40 | Anadromous | Adult |
|  | 48.43564 | 221.89 | Canada | Salmonidae | *Salmo salar* | Summer | River | No evidence | 16.9 |  | Anadromous | Adult |
|  | 48.43564 | 221.89 | Canada | Salmonidae | *Salmo salar* | Summer | River | Cool | 19.0 | 0.42 | Anadromous | Adult |
| Goniea *et al.*, 2006 | 45.93773 | 104.24 | USA | Salmonidae | *Oncorhynchus tshawytscha* | Summer | River | Cool | 23.0 | 4.50 | Anadromous | Adult |
| Goyer *et al.*, 2014 | 46.8026 | 389.84 | Canada | Salmonidae | *Salvelinus fontinalis* | Summer | Lake | Cool |  |  | Resident |  |
| Guzzo *et al.*, 2017 | 49.66666 | 415 | Canada | Salmonidae | *Salvelinus namaycush* | Summer | Lake | Cool | 21 |  | Resident | Adult |
| Hattori and Warburton, 2003 | -26.3333 | 177 | Australia | Melanotaeniidae | *Melanotaenia duboulayi* | Fall | River | Warm |  |  | Resident | Juvenile |
|  | -26.3333 | 177 | Australia | Melanotaeniidae | *Melanotaenia duboulayi* | Fall | River | No evidence |  |  | Resident | Adult |
| Hess *et al.*, 2016 | 45.51279 | 125 | USA | Salmonidae | *Oncorhynchus mykiss* (steelhead) | Summer | River | Cool |  |  | Anadromous | Adult |
| Hitt *et al.*, 2016 | 39.35168 | 145.39 | USA | Salmonidae | *Salvelinus fontinalis* |  | Experiment | Cool | 23.0 | 7.50 |  | Adult |
|  | 39.35168 | 145.39 | USA | Salmonidae | *Salmo trutta* |  | Experiment | Cool | 23.0 | 7.50 |  | Adult |
| Hodge *et al.*, 2017 | 40.17043 | 2189.99 | USA | Salmonidae | *Oncorhynchus clarkii pleuriticus* | Summer | River | Cool | 21.3 |  | Resident |  |
|  | 40.17043 | 2189.99 | USA | Salmonidae | *Oncorhynchus clarkii pleuriticus* | Summer | River | Cool | 27.6 |  | Resident |  |
| Honda *et al.*, 2012 | 43.20791 | 14 | Japan | Salmonidae | *Parahucho perryi* | Summer | River | Cool |  |  | Anadromous | Adult |
|  | 43.20791 | 14 | Japan | Salmonidae | *Parahucho perryi* | Summer | River | No evidence |  |  | Anadromous | Adult |
|  | 43.20791 | 14 | Japan | Salmonidae | *Parahucho perryi* | Summer | River | Cool |  |  | Anadromous | Adult |
| Howell *et al.*, 2010 | 45.55194 | 911.35 | USA | Salmonidae | *Salvelinus confluentus* | Summer | River | No evidence | 20.6 |  | Anadromous | Adult |
|  | 45.55194 | 911.35 | USA | Salmonidae | *Salvelinus confluentus* | Summer | River | No evidence | 20.9 |  | Anadromous | Adult |
| Jackson and Zydlewski, 2009 | 44.77049 | 417 | USA | Salmonidae | *Micropterus dolomieu* | Summer | River | No evidence | 20.2 |  | Resident | Adult |
|  | 44.77049 | 417 | USA | Salmonidae | *Salvelinus fontinalis* | Summer | River | Cool | 20.2 |  | Anadromous | Adult |
|  | 44.77049 | 417 | USA | Salmonidae | *Salmo salar* | Summer | River | Cool | 20.2 |  | Anadromous | Adult |
| Kaeding 2009 | 44.50303 | 2070 | USA | Salmonidae | *Oncorhynchus mykiss* (rainbow) | Summer | River | Cool |  |  |  |  |
|  | 44.50303 | 2070 | USA | Salmonidae | *Salmo trutta* | Summer | River | Cool |  |  |  |  |
| Kaya *et al.*, 1977 | 44.56703 | 2187 | USA | Salmonidae | *Oncorhynchus mykiss* (rainbow) | Summer | River | Cool | 24.0 | 8 |  |  |
|  | 44.56703 | 2187 | USA | Salmonidae | *Salmo trutta* | Summer | River | Cool | 24.0 | 8 |  |  |
| Keefer *et al.*, 2009 | 45.93773 | 104.24 | USA | Salmonidae | *Oncorhynchus mykiss* (steelhead) | Summer | River | Cool | 23.0 | 4.50 | Anadromous | Adult |
| Keefer *et al.*, 2015 | 44.86335 | 39.93 | USA | Salmonidae | *Oncorhynchus tshawytscha* | Summer | River | Cool | 23.7 | 4.50 | Anadromous | Adult |
| Keefer and Caudill, 2016 | 46.59064 | 168.25 | USA | Salmonidae | *Oncorhynchus mykiss* (steelhead) | Summer | River | Cool | 22.4 |  | Anadromous | Adult |
|  | 46.59064 | 168.25 | USA | Salmonidae | *Oncorhynchus mykiss* (steelhead) | Summer | River | Cool | 20.3 |  | Anadromous | Adult |
|  | 46.59064 | 168.25 | USA | Salmonidae | *Oncorhynchus tshawytscha* | Summer | River | Cool | 22.4 |  | Anadromous | Adult |
|  | 46.59064 | 168.25 | USA | Salmonidae | *Oncorhynchus tshawytscha* | Summer | River | Cool | 20.3 |  | Anadromous | Adult |
| Keefer *et al.*, 2018 | 45.93773 | 104.24 | USA | Salmonidae | *Oncorhynchus tshawytscha* | Spring | River | No evidence | 8.0 | 0.40 | Anadromous | Adult |
|  | 45.93773 | 104.24 | USA | Salmonidae | *Oncorhynchus tshawytscha* | Summer | River | No evidence | 22.0 | 0.54 | Anadromous | Adult |
|  | 45.93773 | 104.24 | USA | Salmonidae | *Oncorhynchus tshawytscha* | Summer | River | Cool | 22.0 | 0.54 | Anadromous | Adult |
|  | 45.93773 | 104.24 | USA | Salmonidae | *Oncorhynchus mykiss* (steelhead) | Summer | River | Cool | 22.0 | 6.06 | Anadromous | Adult |
| Keefer *et al.*, 2019 | 45.93773 | 104.24 | USA | Salmonidae | *Oncorhynchus tshawytscha* | Spring | River | No evidence |  |  | Anadromous | Adult |
|  | 45.93773 | 104.24 | USA | Salmonidae | *Oncorhynchus tshawytscha* | Summer | River | No evidence |  |  | Anadromous | Adult |
| Kitagawa *et al.*, 2016 | 39.97096 | 0 | Japan | Salmonidae | *Oncorhynchus keta* | Fall | Coastal area | Cool |  |  | Anadromous | Adult |
| Kristensen *et al.*, 2018 | 56.81714 | 0 | Denmark | Salmonidae | *Salmo trutta* | Spring | Coastal area | Warm |  |  | Anadromous | Adult |
|  | 56.81714 | 0 | Denmark | Salmonidae | *Salmo trutta* | Summer | Coastal area | Cool |  |  | Anadromous | Adult |
| Mackenzie-Grieve and Post, 2006 | 60.49106 | 667.21 | Canada | Salmonidae | *Salvelinus namaycush* | Summer | Lake | Cool |  |  |  | Adult |
|  | 60.58022 | 709.27 | Canada | Salmonidae | *Salvelinus namaycush* | Summer | Lake | No evidence |  |  |  | Adult |
| Matsubu and Simenstad, 2017 | 38.4653 | 8.23 | USA | Salmonidae | *Oncorhynchus mykiss* (steelhead) | Summer | River | No evidence |  |  | Anadromous | Juvenile |
| Matthews *et al.*, 1994 | 39.25389 | 1603 | USA | Salmonidae | *Oncorhynchus mykiss* (rainbow) | Summer | River | No evidence |  |  |  | Adult |
|  | 39.25389 | 1603 | USA | Salmonidae | *Salmo trutta* | Summer | River | No evidence |  |  |  | Adult |
| Moore *et al.*, 2012 | 55.13216 | 103.94 | UK | Salmonidae | *Salmo salar* | Summer | River | Cool | 24.0 | 2.00 | Anadromous | Adult |
| Muhlfeld and Giersch, 2012 | 47.48350 | 845 | USA | Salmonidae | *Salvelinus namaycush* | Summer | River | Cool |  |  | Resident | Adult |
| Newell and Quinn, 2005 | 47.61667 | 2.13 | USA | Salmonidae | *Oncorhynchus nerka* | Fall | Lake | Cool | 25.8 |  | Anadromous | Adult |
| Nielsen and Lisle, 1994 | 39.81433 | 429 | USA | Salmonidae | *Oncorhynchus mykiss* (steelhead) | Summer | River | Cool | 29.0 | 3.50 | Anadromous | Adult |
| Nordahl *et al.*, 2018 | 56.71007 | 0 | Sweden | Cyprinidae | *Cyprinus carpio* | Summer | Experiment | Warm |  | 4.00 | Resident | Adult |
| Nordahl *et al.*, 2020 | 56.63481 | 4 | Sweden | Esocidae | *Esox lucius* | Summer | Coastal area | Warm |  | 0.63 | Anadromous | Adult |
|  | 56.63481 | 4 | Sweden | Esocidae | *Esox lucius* | Winter | Experiment | Warm |  | 0.79 | Anadromous | Adult |
| Peat *et al.*, 2016 | 43.6356 | 0 | Canada | Esocidae | *Esox lucius* | Winter | Coastal area | Warm | 26.2 | 0.70 | Resident | Adult |
|  | 43.6356 | 0 | Canada | Esocidae | *Esox lucius* | Summer | Coastal area | Cool | 26.2 | 0.40 | Resident | Adult |
|  | 43.6356 | 0 | Canada | Centrarchidae | *Micropterus salmoides* | Winter | Coastal area | Warm | 26.2 | 0.90 | Resident | Adult |
|  | 43.6356 | 0 | Canada | Centrarchidae | *Micropterus salmoides* | Summer | Coastal area | Warm | 26.2 | 0.70 | Resident | Adult |
| Petty *et al.*, 2012 | 38.48579 | 1160.98 | USA | Salmonidae | *Salvelinus fontinalis* | Summer | River | Cool |  |  | Anadromous | Adult |
|  | 38.48579 | 1160.98 | USA | Salmonidae | *Salvelinus fontinalis* | Fall | River | No evidence |  |  | Anadromous | Adult |
|  | 38.48579 | 1160.98 | USA | Salmonidae | *Salmo trutta* | Fall | River | No evidence |  |  | Anadromous | Adult |
|  | 38.48579 | 1160.98 | USA | Salmonidae | *Salmo trutta* | Summer | River | Cool |  |  | Anadromous | Adult |
| Plumb and Blanchfield,2009 | 49.66667 | 415 | Canada | Salmonidae | *Salvelinus namaycush* | Summer | Lake | Cool |  |  | Resident | Adult |
| Ritter *et al.*, 2020 | 46.94175 | 1277.72 | USA | Salmonidae | *Prosopium williamsoni* | Summer | River | Cool |  | 2.90 | Anadromous | Adult |
|  | 46.94175 | 1277.72 | USA | Salmonidae | *Salmo trutta* | Summer | River | Cool |  | 2.90 | Anadromous | Adult |
|  | 46.94175 | 1277.72 | USA | Salmonidae | *Oncorhynchus mykiss* (rainbow) | Summer | River | Cool |  | 2.90 | Resident | Adult |
| Rowe and Chisnall, 1995 | -38.0412 | 283 | New Zealand | Salmonidae | *Oncorhynchus mykiss* (rainbow) | Summer | River | Warm |  |  | Resident | Juvenile |
|  | -38.0412 | 283 | New Zealand | Salmonidae | *Oncorhynchus mykiss* (rainbow) | Summer | River | No evidence |  |  | Resident | Adult |
| Schrank *et al.*, 2003 | 42.38286 | 2028.14 | USA | Salmonidae | *Oncorhynchus clarki utah* | Summer | River | No evidence | 27.1 |  | Resident | Adult |
|  | 42.38286 | 2028.14 | USA | Salmonidae | *Oncorhynchus clarki utah* | Summer | River | No evidence | 26.0 |  | Resident | Adult |
| Snucins *et al.*, 1995 | 46.91355 | 324 | Canada | Salmonidae | *Salvelinus namaycush* | Summer | Lake | Cool |  |  | Resident | Adult |
|  | 47.28292 | 400 | Canada | Salmonidae | *Salvelinus namaycush* | Summer | Lake | Cool |  |  | Resident | Adult |
| Spares *et al.*, 2012 | 63.69186 | 0 | Canada | Salmonidae | *Salvelinus alpinus* | Summer | Coastal area | Warm | 14.0 |  | Anadromous | Adult |
| Spigarelli *et al.*, 1983 | 44.28197 | 175 | USA | Salmonidae | *Salmo trutta* |  | Lake | Warm | 17.0 | 5.00 | Resident | Adult |
| Stevens and DuPont, 2011 | 47.65564 | 708.96 | USA | Salmonidae | *Oncorhynchus clarkii lewisi* | Summer | River | Cool |  | 6.00 |  | Adult |
|  | 47.65564 | 708.96 | USA | Salmonidae | *Prosopium williamsoni* | Summer | River | Cool |  | 6.00 |  | Adult |
|  | 47.65564 | 708.96 | USA | Salmonidae | *Oncorhynchus mykiss* (rainbow) | Summer | River | Cool |  | 6.00 |  | Adult |
| Strange 2013 | 41.18592 | 91 | USA | Salmonidae | *Oncorhynchus tshawytscha* | Summer | River | No evidence |  |  | Anadromous | Adult |
| Sutton *et al.*, 2007 | 41.8693 | 533.1 | USA | Salmonidae | *Oncorhynchus kisutch* | Summer | River | Cool | 26.0 | 3.25 | Resident | Juvenile |
|  | 41.8693 | 533.1 | USA | Salmonidae | *Oncorhynchus tshawytscha* | Summer | River | Cool | 26.0 | 3.25 | Resident | Juvenile |
|  | 41.8693 | 533.1 | USA | Salmonidae | *Oncorhynchus mykiss* (steelhead) | Summer | River | Cool | 26.0 | 3.25 | Resident | Juvenile |
|  | 41.8693 | 533.1 | USA | Salmonidae | *Oncorhynchus kisutch* | Summer | River | Cool | 24.0 | 3.25 | Resident | Juvenile |
|  | 41.8693 | 533.1 | USA | Salmonidae | *Oncorhynchus tshawytscha* | Summer | River | Cool | 24.0 | 3.25 | Resident | Juvenile |
|  | 41.8693 | 533.1 | USA | Salmonidae | *Oncorhynchus mykiss* (steelhead) | Summer | River | Cool | 24.0 | 3.25 | Resident | Juvenile |
| Swanberg 1997 | 46.94989 | 1124 | USA | Salmonidae | *Salvelinus confluentus* | Summer | River | Cool |  |  | Resident | Adult |
| Tanaka *et al.*, 2000 | 39.33615 | 0 | Japan | Salmonidae | *Oncorhynchus keta* | Fall | Coastal area | Cool | 14.5 |  | Anadromous | Adult |
|  | 39.33615 | 0 | Japan | Salmonidae | *Oncorhynchus keta* | Fall | Coastal area | Cool | 18.5 | 3.60 | Anadromous | Adult |
|  | 39.33615 | 0 | Japan | Salmonidae | *Oncorhynchus keta* | Fall | Coastal area | Cool | 15.9 | 2.70 | Anadromous | Adult |
|  | 39.33615 | 0 | Japan | Salmonidae | *Oncorhynchus keta* | Fall | Coastal area | Cool | 13.1 |  | Anadromous | Adult |
|  | 39.33615 | 0 | Japan | Salmonidae | *Oncorhynchus keta* | Fall | Coastal area | Cool | 17.3 | 3.15 | Anadromous | Adult |
| Tiffan *et al.*, 2009 | 46.56155 | 224.03 | USA | Salmonidae | *Oncorhynchus tshawytscha* | Summer | Reservoir | Cool | 23.3 | 1.10 |  | Juvenile |
|  | 46.56155 | 224.03 | USA | Salmonidae | *Oncorhynchus tshawytscha* | Summer | Reservoir | Cool | 21.0 | 1.10 |  | Juvenile |
|  | 46.56155 | 224.03 | USA | Salmonidae | *Oncorhynchus tshawytscha* | Summer | Reservoir | Warm | 23.3 | 1.60 |  | Juvenile |
|  | 46.56155 | 224.03 | USA | Salmonidae | *Oncorhynchus tshawytscha* | Summer | Reservoir | Warm | 21.0 | 1.60 |  | Juvenile |
| Venard and Scarnecchia, 2005 | 48.70175 | 746 | USA | Salmonidae | *Salvelinus namaycush* | Summer | Lake | Cool |  |  |  |  |
| Wang *et al.*, 2020 | 39.7297 | 437.69 | USA | Salmonidae | *Oncorhynchus mykiss* (steelhead) | Summer | River | Cool | 21.6 | 2.80 |  | Juvenile |
|  | 39.83847 | 244.45 | USA | Salmonidae | *Oncorhynchus mykiss* (steelhead) | Summer | River | Cool | 26.1 | 6.30 |  | Juvenile |
| Ward *et al.*, 2010 | 52.50958 | 69 | UK | Gasterosteidae | *Gasterosteus aculeatus* | Fall | Experiment | Warm |  |  | Resident | Juvenile |
|  | 52.50958 | 69 | UK | Gasterosteidae | *Gasterosteus aculeatus* | Fall | Experiment | No evidence |  |  | Resident | Adult |
|  | 52.50958 | 69 | UK | Cyprinidae | *Phoxinus phoxinus* | Fall | Experiment | Warm |  |  | Resident | Juvenile |
|  | 52.50958 | 69 | UK | Cyprinidae | *Phoxinus phoxinus* | Fall | Experiment | No evidence |  |  | Resident | Adult |
| Westhoff *et al.*, 2016 | 37.1502 | 212.45 | USA | Centrarchidae | *Micropterus dolomieu* | Spring | River | Cool | 15.7 |  | Anadromous | Adult |
|  | 37.1502 | 212.45 | USA | Centrarchidae | *Micropterus dolomieu* | Summer | River | Cool | 22.2 |  | Anadromous | Adult |
|  | 37.1502 | 212.45 | USA | Centrarchidae | *Micropterus dolomieu* | Fall | River | Warm | 32.0 |  | Anadromous | Adult |
|  | 37.1502 | 212.45 | USA | Centrarchidae | *Micropterus dolomieu* | Winter | River | Warm | 27.5 |  | Anadromous | Adult |
| White *et al.*, 2019 | 39.35168 | 145.39 | USA | Salmonidae | *Salvelinus fontinalis* |  | Experiment | Cool | 23.0 |  | Resident | Juvenile |
| Wilbur *et al.*, 2020 | 46.36938 | 86.87 | Canada | Salmonidae | *Salvelinus fontinalis* | Summer | River | Cool | 28.0 |  | Resident | Juvenile |
|  | 46.36938 | 86.87 | Canada | Salmonidae | *Salvelinus fontinalis* | Summer | River | Cool | 28.0 |  | Anadromous | Adult |
|  | 46.36938 | 86.87 | Canada | Salmonidae | *Salmo salar* | Summer | River | No evidence | 28.0 |  | Resident | Juvenile |
| Zimmerman *et al.*, 1989 | 33.27423 | 63 | USA | Centrarchidae | *Micropterus salmoides* | Fall | Reservoir | Cool | 30.2 |  | Resident | Adult |

**Table S3.** Description of the variables included in the database.

|  | **Variable name** | **Description** | **Values/Levels** |
| --- | --- | --- | --- |
| 1 | Paper | Name and number | Name of first author and year of publication |
| 2 | Lat | Latitude | Value of latitude |
| 3 | Long | Longitude | Value of longitude |
| 4 | Altitude | Altitude | Meters about sea level |
| 5 | Area | Place where the study was carried out | City/County name |
| 6 | Country | Country | Country name |
| 7 | Climate | Climate area of the study | Temperate, tropical or polar |
| 8 | WildExp | Nature condition of the study | Wild or experiment |
| 9 | Family | Taxonomic name of the Family of the species | Name of the family |
| 10 | Native | Native condition of the studied species | Native or non-native |
| 11 | Species | Taxonomic name of the species | Name of the specie |
| 12 | Name | Common name of the species | Name of the specie |
| 13 | NumIndTag | Number of individuals tagged | Number |
| 14 | NumIndTrack | Number of individuals tracked | Number |
| 15 | Year | Year in which the study was carried out | Year |
| 16 | Season | Season in which the study was carried out | Summer, Fall, Winter or Spring |
| 17 | Habitat | Habitat type | River, Lake, Reservoir, Experiment or Coastal area |
| 18 | PointBasin | In studies in river, level of area surveyed | Point (0-5 Km), intermediate (6-15 Km) or Basin (more than 16 Km) |
| 19 | TagMethod | Method used to track the individuals | Radio tag, pit tag, acoustic tag, snorkel and other |
| 20 | TempLogger | Location of temperature recorder | Internal or external |
| 21 | WaterTempRec | Method used to record water temperature | HOBO, VEMCO, probes, IBUTTON and other |
| 22 | Refuse | Use or not use of behavioural thermoregulation | Use or no use |
| 23 | TypeRefuge | Type of thermal refuge used for behavioural thermoregulation | Tributary, spring waters, cool or warm waters, hypolimnion, etc |
| 24 | Thabitatmin | Minimum temperature in the main habitat | Number |
| 25 | Thabitatmax | Maximum temperature in the main habitat | Number |
| 26 | Thabitatmean | Mean temperature in the main habitat | Number |
| 27 | Trefugemin | Minimum temperature in the thermal refuge | Number |
| 28 | Trefugemax | Maximum temperature in the thermal refuge | Number |
| 29 | Trefugemean | Mean temperature in the thermal refuge | Number |
| 30 | Tfishmin | Minimum temperature in the fish body | Number |
| 31 | Tfishmax | Maximum temperature in the fish body | Number |
| 32 | Tfishmean | Mean temperature in the fish body | Number |
| 33 | Tdiffermin | Minimum value of temperature difference between main habitat and thermal refuge | Number |
| 34 | Tdiffermax | Maximum value of temperature difference between main habitat and thermal refuge | Number |
| 35 | Tempdifference | Mean value of temperature difference between main habitat and thermal refuge | Number |
| 36 | Thermoregulation | Direction of behavioural thermoregulation | Cool, warm or no evidence |
| 37 | Migrating status | Migration status of the species | Resident or anadromous |
| 38 | Life stage | Life stage of the studied individuals | Juvenile or adult |
| 39 | Totalweightmean | Total weight of the studied individuals | Number |
| 40 | Lengthmin | Minimum length (fork or total) of the studied individuals | Number |
| 41 | Lengthmax | Maximum length (fork or total) of the studied individuals | Number |
| 42 | Lengthmean | Mean length (fork or total) of the studied individuals | Number |

**Table S4.** List of species and number of observations from all studies reviewed (77 studies) (Full data set n) and for the dataset used for statistical analysis (22 studies) (Cool data set n).

| **Family** | **Species** | **Full dataset n** | **Cool data set n** |
| --- | --- | --- | --- |
| Esocidae | *Esox lucius* | 5 |  |
| Catostomidae | *Moxostoma duquesnei* | 1 |  |
| Centrarchidae | *Micropterus dolomieu* | 9 |  |
|  | *Micropterus salmoides* | 4 |  |
|  | *Lepomis macrochirus* | 1 |  |
| Cottidae | *Uranidea bairdii* | 2 |  |
|  | *Uranidea cognata* | 2 |  |
| Cyprinidae | *Cyprinus carpio* | 3 |  |
|  | *Phoxinus phoxinus* | 2 |  |
| Gasterosteidae | *Gasterosteus aculeatus* | 2 |  |
| Melanotaeniidae | *Melanotaenia duboulayi* | 2 |  |
| Salmonidae | *Oncorhynchus clarkii utah* | 2 |  |
|  | *Oncorhynchus clarkii lewisi* | 3 | 3 |
|  | *Oncorhynchus clarkii pleuriticus* | 2 |  |
|  | *Oncorhynchus keta* | 7 |  |
|  | *Oncorhynchus kisutch* | 14 | 8 |
|  | *Oncorhynchus mykiss* | 36 | 22 |
|  | *Oncorhynchus mykiss irideus* | 3 | 3 |
|  | *Oncorhynchus nerka* | 4 | 1 |
|  | *Oncorhynchus tshawytscha* | 33 | 16 |
|  | *Parahucho perryi* | 3 |  |
|  | *Prosopium williamsoni* | 2 | 2 |
|  | *Salmo salar* | 21 | 4 |
|  | *Salmo trutta* | 10 | 2 |
|  | *Salvelinus alpinus* | 1 |  |
|  | *Salvelinus confluentus* | 3 |  |
|  | *Salvelinus fontinalis* | 21 | 2 |
|  | *Salvelinus namaycush* | 10 |  |

**Table S5.** Fish detection methods extracted from the papers (second column), their number of entries (N obs) and their percentage (%). Column five groups the different methods according to their typology.

|  | **Tagging Method/ Fish detection** | **N obs** | **%** | **Grouping** | **N obs** | **%** |
| --- | --- | --- | --- | --- | --- | --- |
| 1 | RDST | 13 | 6.25 | Radio Tag | 74 | 35.58 |
| 2 | Radio tag | 38 | 18.27 |  |  |  |
| 3 | Temperature-sensitive Radio tag | 20 | 9.62 |  |  |  |
| 4 | Radio and archival tag | 2 | 0.96 |  |  |  |
| 5 | Radiotransmiters | 1 | 0.48 |  |  |  |
| 6 | Temperature-sensitive Archival tag | 4 | 1.92 | Archival Tag | 4 | 1.92 |
| 7 | Temperature-sensitive Acoustic tag | 2 | 0.96 | Acoustic Tag | 25 | 12.02 |
| 8 | Acoustic tag | 23 | 11.06 |  |  |  |
| 9 | DST | 4 | 1.92 | Undefined Tag | 14 | 6.73 |
| 10 | DST and PIT tag | 2 | 0.96 |  |  |  |
| 11 | Micro data loggers | 5 | 2.40 |  |  |  |
| 12 | Microprocessor-based data logger | 1 | 0.48 |  |  |  |
| 13 | Temperature-sensing ultrasonic transmiters | 2 | 0.96 |  |  |  |
| 14 | PIT tag | 16 | 7.69 | PIT tag | 16 | 7.69 |
| 15 | Snorkel | 49 | 23.56 | Visual detection | 67 | 32.21 |
| 16 | Sampling | 4 | 1.92 |  |  |  |
| 17 | Visual survey | 4 | 1.92 |  |  |  |
| 18 | High-resolution camera | 5 | 2.40 |  |  |  |
| 19 | Underwater videography | 1 | 0.48 |  |  |  |
| 20 | Electrofishing | 2 | 0.96 |  |  |  |
| 21 | Angling | 1 | 0.48 |  |  |  |
| 22 | Nets | 1 | 0.48 |  |  |  |
| 23 | Petersen discs | 2 | 0.96 | External marks | 4 | 1.92 |
| 24 | Plastic disks | 2 | 0.96 |  |  |  |
| 25 | Echosounder | 4 | 1.92 | Echosounder | 4 | 1.92 |

**Table S6.** Water temperature recording methods extracted from the papers (second column), their number of entries (N obs) and their percentage (%). Column five groups the different methods according to their typology.

|  | **Temperature water recorder** | **N obs** | **%** | **Grouping** | **N obs** | **%** |
| --- | --- | --- | --- | --- | --- | --- |
| 1 | HOBO | 65 | 31.25 | Temperature loggers | 127 | 61.06 |
| 2 | IBUTTON | 14 | 6.73 |  |  |  |
| 3 | VEMCO | 3 | 1.44 |  |  |  |
| 4 | iBCod temperature data loggers | 6 | 2.88 |  |  |  |
| 5 | Temperature loggers | 14 | 6.73 |  |  |  |
| 6 | Onset temperature loggers | 2 | 0.96 |  |  |  |
| 7 | Tidbit temperature data loggers | 4 | 1.92 |  |  |  |
| 8 | Data loggers | 2 | 0.96 |  |  |  |
| 9 | Thermistors and transmiters | 3 | 1.44 |  |  |  |
| 10 | Electronic thermistor | 2 | 0.96 |  |  |  |
| 11 | Onset ProV2 | 1 | 0.48 |  |  |  |
| 12 | iBCod tags | 1 | 0.48 |  |  |  |
| 13 | Thermographs | 6 | 2.88 |  |  |  |
| 14 | Digital thermograph and thermocouple | 2 | 0.96 |  |  |  |
| 15 | Stowaway or Tidbit thermographs | 2 | 0.96 |  |  |  |
| 16 | HYDROLAB sonde | 1 | 0.48 | Thermometers | 48 | 23.08 |
| 17 | Handheld meter | 13 | 6.25 |  |  |  |
| 18 | Datasonde | 2 | 0.96 |  |  |  |
| 19 | Digital probe | 4 | 1.92 |  |  |  |
| 20 | Thermometer | 7 | 3.37 |  |  |  |
| 21 | Visipak VIP501 RTD digital temperature indicator and probes | 1 | 0.48 |  |  |  |
| 22 | YSI field DO/BDO meter | 2 | 0.96 |  |  |  |
| 23 | YSI handheld | 12 | 5.77 |  |  |  |
| 24 | YSI probe | 6 | 2.88 |  |  |  |
| 25 | Bathythermograph | 6 | 2.88 | Bathythermograph | 6 | 2.88 |
| 26 | Monitoring Station | 4 | 1.92 | Monitoring Station | 4 | 1.92 |
| 27 | Micro data loggers | 5 | 2.40 | Inside the fish | 23 | 11.06 |
| 28 | DSTs | 1 | 0.48 |  |  |  |
| 29 | Internal | 17 | 8.17 |  |  |  |

**Table S7.** Descriptive statistics of the multiple linear regression models quantifying relationships between temperature difference and latitude and altitude. The Akaike information criterion of the models is shown to compare the different models (* p<0.05).

| **Model** | **Coefficient** | **SE** | **t value** | **p** | **df** | **p** | **R square** | **AIC value** |
| --- | --- | --- | --- | --- | --- | --- | --- | --- |
| Intercept  Latitude | -0.2199  -0.9758 | 0.1167  0.2055 | -1.885  -4.748 | 0.064  <0.001* | 58 | <0.001* | 0.27 | 151.89 |
| Intercept  Latitude  Altitude | -0.21898  -0.97497  0.01823 | 0.11806  0.20748  0.18726 | -1.855  -4.699  -0.097 | 0.069  <0.001*  0.923 | 57 | <0.001* | 0.25 | 153.88 |
| Intercept  Latitude  Altitude  Latitude : Altitude | -0.2093  -0.9542  -0.1886  0.5377 | 0.1189  0.2093  0.2724  0.6230 | -1.761  -4.559  -0.692  0.863 | 0.084  <0.001*  0.492  0.392 | 56 | <0.001* | 0.25 | 155.09 |

**Table S8.** Comparisons between linear mixed-effects models relating variation in temperature difference to the maximum habitat temperature (Thabitatmax) and life stage. The Akaike information criterion of the models is shown. Estimates of coefficients, standard error (ES) (fixed effects) and their standard deviation across species (random effects) are indicated (* p<0.05).

| **Model** | **Fixed effects Effect** | | | | **Random effects (\|Species) SD** | **AIC value** |
| --- | --- | --- | --- | --- | --- | --- |
|  | **Coefficient** | **SE** | **df** | **p** |  |  |
| Intercept  Thabitatmax | -0.7338  0.5800 | 0.3019  0.3728 | 6.73  34.00 | 0.047*  0.129 | 0.6683 | 93.77 |
| Intercept  Thabitatmax  Juvenile | -0.8231  0.4619  0.3673 | 0.2891  0.3777  0.3067 | 7.72  32.64  32.86 | 0.022*  0.230  0.240 | 0.5681 | 95.07 |
| Intercept  Thabitatmax  Juvenile  Thabitatmax : Juvenile | -0.73547  -0.13129  -0.08866  2.25276 | 0.25724  0.39438  0.31933  0.76960 | 8.45  29.67  31.78  30.36 | 0.020*  0.742  0.783  0.006* | 0.4932 | 87.95 |

**Appendix S1**

**Annex A: Outliers detection**

Data explorations were performed between temperature difference with latitude and altitude variables.

We performed the function “influence.measures(model, infl = influence(model))” in r. This function produces a class "infl" object tabular display showing the DFBETAS for each model variable, DFFITS, covariance ratios, Cook's distances and the diagonal elements of the hat matrix. These functions provide direct access to the corresponding diagnostic quantities by marking influential cases with an asterisk.

We then removed from the database the entries (studies) that were marked with asterisks and ran the regression model again without these entries. We compared the initial model with the new model and selected the model with the lowest AIC value.

**Altitude:**

Output of influence.measures(initialmodel, infl = influence(initialmodel)):

| **Cases** | **Dfbetas** | **Dffits** | **Covariance ratios** | **Cook's distances** | **Hat** |
| --- | --- | --- | --- | --- | --- |
| 62 | 0.56 | 0.58 | 1.40 | 0.17 | 0.28 * |
| 63 | 0.56 | 0.58 | 1.40 | 0.17 | 0.28 * |

The influential data correspond to Kaya et al. 1977, which presents a much higher altitude value than the rest of the studies and a higher temperature difference value.

Comparison between the initial model and the new model without the entries from Kaya et al. 1977:

|  |  | **Estimate** | **SE** | **p** | **F** | **Df** | **r** | **p** | **AIC** |
| --- | --- | --- | --- | --- | --- | --- | --- | --- | --- |
| **Initial model** | |  |  |  |  |  |  |  |  |
|  | Intercept | -0.0016 | 0.1261 | 0.990 | 3.245 | 61 | 0.03495 | 0.077 | 181 |
|  | Altitude | 0.2621 | 0.1455 | 0.077 |  |  |  |  |  |
| **New model** | |  |  |  |  |  |  |  |  |
|  | Intercept | -0.0197 | 0.12574 | 0.876 | 0.0009741 | 59 | -0.01693 | 0.975 | 174 |
|  | Altitude | -0.0067 | 0.21533 | 0.975 |  |  |  |  |  |

**Latitude:**

Output of influence.measures(initialmodel, infl = influence(initialmodel)):

| **Cases** | **Dfbetas** | **Dffits** | **Covariance ratios** | **Cook's distances** | **Hat** |
| --- | --- | --- | --- | --- | --- |
| 55 | 0.58 | 0.60 | 1.36 | 0.18 | 0.26 * |

The influential data corresponded to Moore et al. 2012, which presents a much higher latitude value than the rest of the studies included in the data set for this analysis (63 observations from 22 studies).

Comparison between the initial model and the new model without the entries from Moore et al. 2012:

|  |  | **Estimate** | **SE** | **p** | **F** | **Df** | **r** | **p** | **AIC** |
| --- | --- | --- | --- | --- | --- | --- | --- | --- | --- |
| **Initial model** | |  |  |  |  |  |  |  |  |
|  | Intercept | -0.1152 | 0.1164 | 0.326 | 19.35 | 61 | 0.2284 | <0.001* | 167 |
|  | Latitude | -0.8306 | 0.1888 | <0.001* |  |  |  |  |  |
| **New model** | |  |  |  |  |  |  |  |  |
|  | Intercept | -0.1513 | 0.1219 | 0.220 | 18.52 | 60 | 0.2232 | <0.001* | 164 |
|  | Latitude | -0.9398 | 0.2184 | <0.001* |  |  |  |  |  |

Comparison between the final complete models:

|  |  | **Estimate** | **SE** | **p** | **F** | **Df** | **r** | **p** | **AIC** |
| --- | --- | --- | --- | --- | --- | --- | --- | --- | --- |
| **Model with all the entries** | | | | | | | | | |
|  | Intercept | -0.0179 | 0.1078 | 0.871 | 8.997 | 59 | 0.279 | <0.001* | 164 |
|  | Latitude | -1.6156 | 0.3423 | <0.001* |  |  |  |  |  |
|  | Altitude | 0.4606 | 0.1854 | 0.016* |  |  |  |  |  |
|  | Lat : Alt | -0.9997 | 0.6795 | 0.147 |  |  |  |  |  |
| **Model without Kaya et al. 1977 entries** | | | | | | | | | |
|  | Intercept | -0.0237 | 0.1090 | 0.829 | 7.384 | 57 | 0.242 | <0.001* | 158 |
|  | Latitude | -1.4664 | 0.3505 | <0.001* |  |  |  |  |  |
|  | Altitude | -0.0307 | 0.3403 | 0.929 |  |  |  |  |  |
|  | Lat : Alt | -0.0968 | 0.8551 | 0.910 |  |  |  |  |  |
| **Model without Moore et al. 2012 entries** | | | | | | | | | |
|  | Intercept | -0.0247 | 0.1109 | 0.824 | 8.394 | 58 | 0.2667 | <0.001* | 162 |
|  | Latitude | -1.6900 | 0.3640 | <0.001* |  |  |  |  |  |
|  | Altitude | 0.4196 | 0.1975 | 0.038* |  |  |  |  |  |
|  | Lat : Alt | -0.6939 | 0.8395 | 0.412 |  |  |  |  |  |
| **Model without Kaya et al. 1977 and Moore et al. 2012 entries** | | | | | | | | | |
|  | Intercept | -0.0425 | 0.1086 | 0.698 | 7.605 | 56 | 0.2514 | <0.001* | 155 |
|  | Latitude | -1.5952 | 0.3586 | <0.001* |  |  |  |  |  |
|  | Altitude | -0.3058 | 0.3875 | 0.433 |  |  |  |  |  |
|  | Lat : Alt | 0.9723 | 1.1266 | 0.392 |  |  |  |  |  |
